# Preparation of Curcumin Nanocomposite Drug Delivery System and Its Therapeutic Efficacy on Skin Injury

**DOI:** 10.3390/gels11090727

**Published:** 2025-09-11

**Authors:** Ye Jin, Yuzhou Liu, Ying Wang, Xintong Liu, Qixuan Yu, Da Liu, Ning Cui

**Affiliations:** 1 Northeast Asian Institute of Traditional Chinese Medicine, Changchun University of Chinese Medicine, Changchun 130117, China; 2 College of Integrated Traditional Chinese and Western Medicine, Changchun University of Chinese Medicine, Changchun 130117, China; 3 Public Experimental Center, Changchun University of Chinese Medicine, Changchun 130117, China; 4 College of Pharmacy, Changchun University of Chinese Medicine, Changchun 130117, China

**Keywords:** *Curcumin*, gel, micelles, microneedling, skin damage

## Abstract

**Background:** Skin injuries, such as chronic wounds and inflammatory skin diseases, often face limitations in treatment efficacy due to the low efficiency of transdermal drug delivery and insufficient local concentrations. *Curcumin* (CUR), a natural compound with anti-inflammatory and antioxidant properties, has demonstrated potential in the repair of skin damage; however, its clinical application is hindered by its physicochemical characteristics. This study constructs a novel nanocomposite drug delivery system: CUR-loaded micellar nanocomposite gel (CUR-M-DMNs-Gel). A composite system is used to achieve the efficient solubilization and enhanced transdermal permeation of *CUR*, thereby providing a novel formulation approach for the treatment of skin diseases. **Methods:** CUR-loaded micellar (CUR-M) utilizes CUR as the core active ingredient, which possesses multiple pharmacological effects including anti-inflammatory and antioxidant properties. TPGS serves as a micellar carrier that not only enhances the solubility and stability of CUR through its amphiphilic structure but also facilitates drug absorption and transport within the body. In dissolvable microneedles (DMNs), PVP K30 forms a stable three-dimensional network structure through entanglement of polymer chains, ensuring sufficient mechanical strength for effective penetration of the skin barrier. Meanwhile, PVP K90, with its higher molecular weight, enhances the backing’s support and toughness to prevent needle breakage during application. The incorporation of hyaluronic acid (HA) improves both the moisture retention and adhesion properties at the needle tips, ensuring gradual dissolution and release of loaded CUR-M within the skin. In CUR-loaded micellar gel (CUR-M-Gel), PVP K30 increases both adhesive and cohesive forces in the gel through chain entanglement and hydrogen-bonding interactions. Tartaric acid precisely regulates pH levels to adjust crosslinking density; glycerol provides a long-lasting moisturizing environment for the gel; aluminum chloride enhances mechanical stability and controlled drug-release capabilities; NP-700 optimizes dispersion characteristics and compatibility within the system. **Results:** In vitro experiments demonstrated that the CUR-M-DMNs-Gel composite system exhibited enhanced transdermal penetration, with a cumulative transdermal efficiency significantly surpassing that of single-component formulations. In the mouse skin defect model, CUR-M-DMNs-Gel facilitated collagen deposition and effectively inhibited the expression of inflammatory cytokines (TNF-α, IL-6, and IL-1β). In the mouse skin photoaging model, CUR-M-DMNs-Gel markedly reduced dermal thickness, alleviated damage to elastic fibers, and suppressed inflammatory responses. **Conclusions:** The CUR-M-DMNs-Gel system can enhance wound healing through subcutaneous localization, achieving long-term sustained efficacy. This innovative approach offers new insights into the treatment of skin injuries.

## 1. Introduction

With the enhancement of living standards and increasing awareness of health, skin health issues have received growing attention. Among various skin conditions, skin damage can be broadly classified into two major categories: photoaging and wound injury. Skin aging is a complex biological process involving both endogenous and exogenous mechanisms [1]. Endogenous aging arises from intrinsic, irreversible factors such as physiological aging processes, whereas exogenous aging is primarily induced by external environmental influences, including ultraviolet radiation, exposure to chemical agents, and cigarette smoking [2,3]. Among these conditions, photoaging resulting from prolonged exposure to ultraviolet radiation is particularly prevalent, with clinical manifestations such as rough skin texture, loss of elasticity, the formation of deep wrinkles, irregular pigmentation, and, in severe cases, the potential induction of skin cancer [4]. Skin damage resulting from photoaging can be managed through the topical administration of retinoic acid derivatives, antioxidants, and other therapeutic agents [5,6]. On the other hand, wound injury, as a prevalent clinical concern, may result in infection or even pose life-threatening risks if not appropriately managed. While traditional dressings are capable of absorbing exudate and preventing microbial invasion, they do not actively promote wound healing [7]. Recent studies have demonstrated that a moderately moist wound environment facilitates cell migration and enhances the formation of granulation tissue, thereby improving wound-healing outcomes [8]. Therefore, conventional wound dressings exhibit significant limitations in facilitating tissue regeneration and associated biological processes [9]. In summary, for the two major categories of skin conditions—photoaging and wound injury—conventional preventive and therapeutic strategies are increasingly inadequate to fulfill clinical demands. The development and implementation of novel technologies and approaches are anticipated to offer more effective and optimized solutions for the management of skin damage [10,11].

Turmeric (*Curcuma longa*), a member of the Zingiberaceae family, is extensively utilized in the food industry as both a flavoring agent and a natural coloring agent [12]. CUR, the primary active constituent of turmeric, has attracted considerable scientific attention due to its notable biological activities [13]. Contemporary research has demonstrated that CUR exhibits notable pharmacological effects, including anti-inflammatory and antioxidant properties [14]. Recent studies have demonstrated that CUR significantly alleviates a range of dermatological conditions [15,16]. In the treatment of cutaneous disorders such as psoriasis, CUR demonstrates therapeutic potential through the modulation of inflammatory responses, among other mechanisms, thereby providing a natural pharmacological option for the management of dermatological diseases [17]. CUR exhibited substantial antioxidative protective effects across various oxidative stress models [18]. In the UV-induced photoaging damage model, CUR exhibited more comprehensive protective effects [19]. CUR has exhibited notable therapeutic efficacy in promoting wound healing through the acceleration of collagen deposition, facilitation of granulation tissue formation, and enhancement of wound contraction [20,21]. The clinical application of CUR has been constrained by its limited water solubility and low stability, despite its possession of antioxidant and anti-inflammatory properties [22,23]. To address this limitation, nanotechnology-based delivery systems, such as lipid-based carriers [24], nanogels [25], and nanoemulsions [26], have been utilized to improve the solubility and stability of CUR [27,28], providing novel insights into its potential application in wound repair [29].

**Figure 1:**
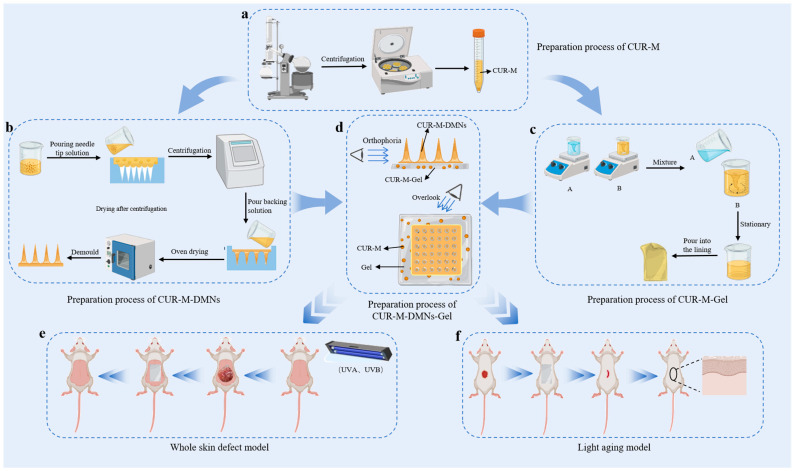
Schematic illustration of CUR-M-DMNs-Gel for skin wound therapy. (**a**) Fabrication process of CUR-M. (**b**) Preparation procedure of CUR-M-DMNs. (**c**) Construction of CUR-M-Gel. (**d**) Frontal and top views of CUR-M-DMNs-Gel. (**e**) Proposed therapeutic mechanism of CUR-M-DMNs-Gel in wound healing. (**f**) Schematic of CUR-M-DMNs-Gel application for photoaging treatment.

This study developed a CUR-M-DMNs-Gel composite delivery system that integrates the solubilization properties of micelles, the transdermal permeation enhancement effects of microneedles, and the adhesion-controlled release functionality of gels. The aim is to improve the transdermal delivery efficiency of CUR. First, CUR-M was prepared by leveraging its hydrophilic shell and hydrophobic core structure [30], which substantially improved drug solubility and stability [31,32] (Figure 1a). To further improve transdermal penetration, CUR-M-DMNs were fabricated [33,34]. These microneedles enable painless transdermal penetration and facilitate rapid CUR release into the dermal tissue [35], while the dissolving microneedles simultaneously establish rapid drug delivery pathways through their dissolution process (Figure 1b). To prolong drug retention time, CUR-M-Gel with sustained-release properties was formulated [36]. CUR-M-Gel not only achieved sustained drug release on the skin surface but also ensured stable retention of CUR-M-DMNs due to its adhesive properties [37] (Figure 1c). To further validate the synergistic therapeutic effects of CUR-M-DMNs-Gel drug delivery system on photoaging and wound repair, in vivo animal experiments were performed. (Figure 1e,f).

The current literature reports on enhancing the bioavailability of CUR micelles loaded in hyaluronic acid (HA) composite microneedles for melanoma treatment. These micelles are constructed using a polymeric system based on quercetin-dithiopropionic acid-oligo-hyaluronic acid (Que-DA-oHA), with the microneedle matrix optimized solely around the ratio of HA to carboxymethyl starch sodium (CMS-Na) [38]. Currently, research has been conducted to develop a CUR carrier through the combination of albumin and encapsulation in extracellular vesicles (EVs), utilizing dissolvable microneedle arrays (dMNAs) for delivery aimed at inhibiting skin inflammation. The core concept lies in leveraging the biocompatibility of EVs along with the stabilizing properties of albumin [39]. The preparation of micelles in the design of drug delivery systems in this study utilizes a combination of CUR and TPGS, with TPGS serving as a safe excipient that possesses both emulsifying and permeation-enhancing properties. The fabrication of microneedles integrates multiple materials, including PVPK30, HA, and PVPK90. Through the synergistic effects of PVP with varying molecular weights, it is possible to achieve an optimal balance between mechanical strength and solubility performance. Moreover, this research innovatively incorporates gel formulations wherein tartaric acid can modulate pH levels to maintain the stability of CUR; glycerin enhances moisture retention; and aluminum chloride may reduce exudate through its astringent properties. NP-700 is employed as a thickening agent to ensure the stability of the formulation.

This study uniquely combines three technological advantages to establish a “micelle-microneedle-gel” multi-formulation collaborative system. This approach not only preserves the rapid transdermal benefits offered by microneedles but also allows gels to function not only as drug carriers but also directly participate in regulating the microenvironment at wound sites.

## 2. Results and Discussion

### 2.1. Characterization of CUR-M, CUR-M-DMNs and CUR-M-Gel

#### 2.1.1. Characterization of CUR-M

**Figure 2:**
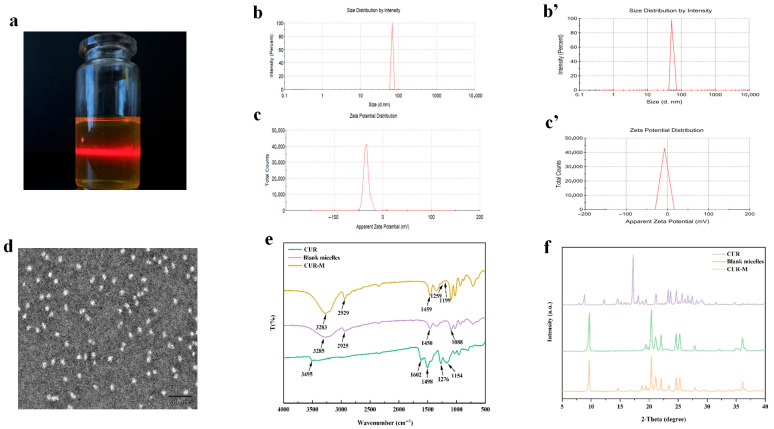
Characterization of CUR-M. (**a**) Tyndall effect of CUR-M. (**b**) Particle size distribution of CUR-M. (**b’**) Particle size distribution of blank micelles. (**c**) Zeta potential of CUR-M. (**c’**) Zeta potential of blank micelles. (**d**) TEM image of CUR-M. (**e**) FTIR spectrum of CUR-M. (**f**) XRD pattern of CUR-M.

The CUR-M solution remained transparent and clear. Upon irradiation with infrared light, a distinct light path was observed within the solution, indicating a pronounced Tyndall effect. This optical phenomenon provides conclusive evidence for the formation of a colloidal system in the solution (Figure 2a).

The CUR-M micelles exhibited an average particle size of 68.70 ± 0.92 nm and a zeta potential of −7.91 ± 1.12 mV, while the blank micelles demonstrated an average particle size of 51.77 ± 0.83 nm and a zeta potential of −7.08 ± 0.92 mV. The PDI of the drug-loaded micelles is [0.15] ± [0.01], while the PDI of the empty micelles is [0.13] ± [0.03] (*n* = 3). The increase in particle size intuitively confirms the successful encapsulation of the hydrophobic drug CUR into the hydrophobic core of micelles, resulting in core expansion. More importantly, both systems exhibit a very low polydispersity index (PDI), with values less than 0.3, indicating that they possess monodispersity and have a highly uniform particle size distribution. This suggests that the drug loading process did not compromise the structural integrity of the micelles, successfully yielding uniformly sized drug-loaded nanoparticles. In terms of surface charge, the similar absolute values of Zeta potential for both systems indicate that the drug loading process maintained stability in surface properties of the micelles. This moderate negative charge contributes to long-term stability within the colloidal system through electrostatic repulsion, preventing aggregation during storage. The drug-loaded micelle system (CUR-M) exhibits subtle differences from the blank micelle system while maintaining high consistency in key physicochemical characteristics. This provides strong evidence for successful drug loading and formation of a stable and homogeneous nanodelivery system (Figure 2b,b’,c,c’). Transmission electron microscopy (TEM) characterization revealed that the CUR-M particles possessed a well-defined spherical morphology without significant aggregation. The structural integrity was well maintained, and a homogeneous dispersion was observed (Figure 2d).

The CUR-M formulation exhibited an EE of 95.36 ± 0.22% and a DL capacity of 5.53 ± 0.04%. During the 21-day stability study conducted at 4 °C, the CUR-M formulation demonstrated excellent physical stability. No visible changes in appearance were observed, and the solution retained its clarity and transparency throughout the entire storage period. Importantly, no flocculent precipitation was detected, indicating that CUR-M possesses good stability under low-temperature storage conditions (Table 1). Fourier-transform infrared (FTIR) spectroscopy analysis revealed characteristic absorption bands corresponding to free CUR at 1276 cm^−1^ (C–H in-plane bending vibration of alkene), 1602 cm^−1^ (C=O stretching vibration), and 1498 cm^−1^ (aromatic C=C stretching vibration), along with a distinct phenolic O–H stretching vibration peak at 3495 cm^−1^. Blank micelles exhibited characteristic absorption peaks at 1450 cm^−1^ (C–H bending vibration), 1088 cm^−1^ (C–O–C stretching vibration), and broad O–H stretching vibrations at 2925 cm^−1^ and 3285 cm^−1^. Notably, in the FTIR spectrum of CUR-M, the characteristic peaks of CUR were shifted to 1459 cm^−1^, 1259 cm^−1^, and 1199 cm^−1^. The original phenolic O–H stretching vibration at 3495 cm^−1^ was no longer present, while a broad hydroxyl vibration peak appeared at 3283 cm^−1^, corresponding to that of blank micelles, and was accompanied by a distinct red shift. These spectral changes provide conclusive evidence for the successful encapsulation of CUR within the micellar carrier through intermolecular interactions (Figure 2e). X-ray diffraction (XRD) analysis revealed characteristic crystalline diffraction peaks of raw CUR at diffraction angles of 8.86°, 14.49°, 17.24°, 18.12°, 21.19°, 23.26°, 24.70°, 25.69°, 27.41°, 28.17°, and 29.04°, confirming its crystalline nature (Figure 2f). Notably, these characteristic diffraction peaks were completely absent in both CUR-M and blank micelles, indicating an amorphous dispersion of CUR within the micellar matrix. This observation constitutes strong evidence supporting the effective incorporation of CUR molecules into the micellar nanocarriers.

#### 2.1.2. Characterization of CUR-M-DMNs

**Figure 3:**
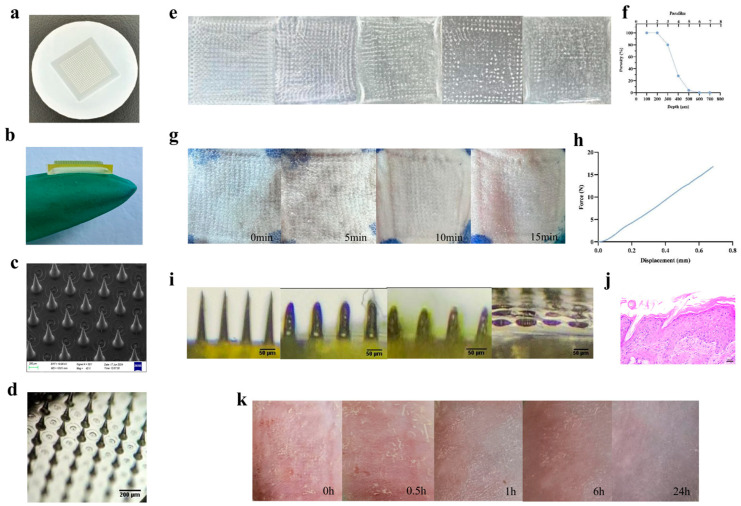
Characterization of CUR-M-DMNs. (**a**,**b**) Schematic diagrams of the mold and the macroscopic appearance of CUR-M-DMNs. (**c**,**d**) SEM images and microscopic views of CUR-M-DMNs. (**e**) Diagram for the assessment of CUR-M-DMNs’ puncture capability. (**f**) The percentage of pores generated by CUR-M-DMNs in each layer of Parafilm membrane. (**g**) Recovery pictures of CUR-M-DMNs over time. (**h**) Force-displacement curve of CUR-M-DMNs. (**i**,**j**) Dissolution pictures of CUR-M-DMNs at different time points and H&E staining of skin penetration at 0 min. (**k**) Skin irritation assessment of CUR-M-DMNs at various time intervals.

The structural design of the mold is presented in Figure 3a. The demolded CUR-M-DMNs exhibited a square-patch configuration with well-aligned microneedle arrays, displaying smooth surface topography and intact morphological integrity without any observable structural defects (Figure 3b). All needle-shaped structures were confirmed to possess a cylindrical morphology based on SEM and optical microscopy observations. The CUR-M-DMNs prepared after demolding are square patches, comprising 400 needles arranged in an orderly array with smooth surfaces and intact shapes, suggests consistent mechanical strength across the entire patch, which is crucial for uniform skin penetration. The needle height of approximately 800 μm is optimal for penetrating the stratum corneum and epidermis to reach the superficial dermis, thereby enabling efficient transdermal delivery of CUR. The square base design with a bottom diameter of 250 μm and an inter-needle spacing of 450 μm ensures sufficient mechanical stability to prevent needle collapse during insertion while minimizing skin deformation and avoiding patch clogging caused by too dense an arrangement. This appropriate spacing also facilitates efficient diffusion of the drug into the surrounding tissue after dissolution (Figure 3c,d). Mechanical testing revealed that the CUR-M-DMNs exhibited a linearly increasing force-displacement profile throughout the loading process, with no abrupt pressure drop detected. This mechanical behavior suggests that the microneedles have sufficient structural integrity to effectively penetrate the stratum corneum barrier (Figure 3e). The puncture test demonstrated that CUR-M-DMNs were capable of penetrating the skin simulant when inserted into the Parafilm membrane under controlled velocity and pressure conditions. Quantitative analysis of perforation efficiency showed a progressively decreasing porosity rate across layers 1 to 5 (100%, 100%, 80%, 28%, and 4%, respectively). These findings provide strong evidence supporting the adequate mechanical strength of CUR-M-DMNs (Figure 3f). The experimental results demonstrated that CUR-M-DMNs exhibited favorable dissolution characteristics following insertion into ex vivo skin models. Due to the hydrophilic nature of hyaluronic acid (HA), complete dissolution of the microneedle system was achieved within 10 min post-insertion, confirming its excellent rapid-dissolution properties (Figure 3g). The cutaneous recovery experiments revealed that although distinct micropore traces were initially observed on murine skin immediately after removal of CUR-M-DMNs, full closure occurred within 15 min, indicating good compliance of the microneedle system (Figure 3h). Dermal irritation testing showed that CUR-M-DMNs induced transient erythema and mild edema in murine epidermal tissue immediately after insertion. However, these localized irritant responses were found to resolve completely within 30 min, with no persistent tissue edema or other pathological changes detected throughout the experimental period, further confirming the favorable biocompatibility of the microneedle system (Figure 3i).

#### 2.1.3. Characterization of CUR-M-Gel

**Figure 4:**
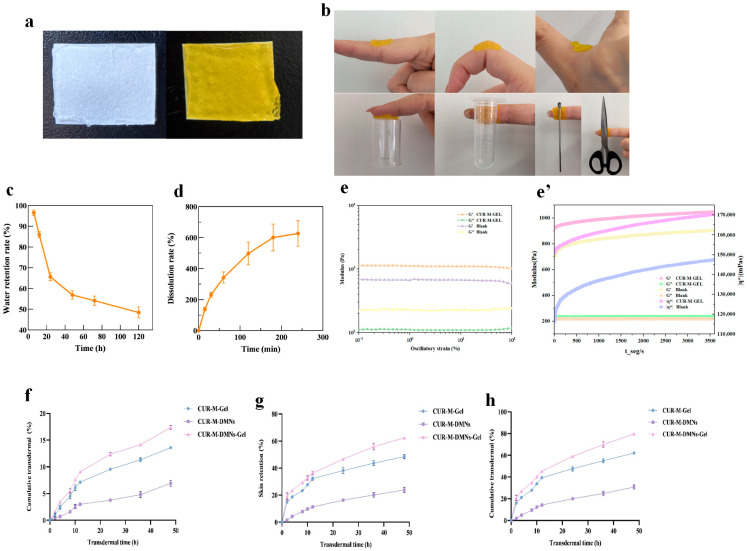
Characterization of CUR-M-Gel. (**a**) Macroscopic appearance of blank Gel and CUR-M-Gel. (**b**) Schematic illustration of CUR-M-Gel adhesion to various surfaces (skin, joints, plastic, glass, iron, and steel). (**c**) Water retention capacity of CUR-M-Gel. (**d**) Swelling behavior of CUR-M-Gel. (**e**) Rheological curves of CUR-M-Gel and blank gel. (**e’**) Time-sweep experiment of CUR-M-Gel and blank gel. (**f**) Cumulative transdermal permeation rate. (**g**) Retention rate. (**h**) Total cumulative transdermal permeation.

Morphological observation revealed that both blank Gel and CUR-M-Gel exhibited a transparent appearance with a uniform and homogeneous texture (Figure 4a). Adhesion testing demonstrated that CUR-M-Gel maintained strong adherence to various substrates, including skin, joints, plastics, glass, iron, and steel, without detachment. Importantly, changes in positioning did not affect its adhesive stability, confirming the material’s excellent adhesion properties (Figure 4b). The water retention test showed that CUR-M-Gel maintained a stable water retention capacity of 48.44% after an initial decline from 96.55% over a 120 h dehydration period, indicating durable hydration preservation capability (Figure 4c). For the swelling test, CUR-M-Gel was immersed in deionized water at room temperature. During the 240 min immersion period, the swelling ratio gradually reached a plateau, demonstrating favorable swelling stability of the material (Figure 4d). Rheological characterization revealed that the storage modulus (G′) of CUR-M-Gel consistently exceeded the loss modulus (G″) across the entire tested frequency range (0.1–100 rad/s), with no crossover point observed between the two moduli. This rheological profile indicated that the gel retained solid-like characteristics without undergoing a gel-sol transition under mechanical stresses spanning low to high-frequency regimes. The absence of structural degradation under oscillatory shear deformation further confirmed the exceptional mechanical stability of CUR-M-Gel, which can be attributed to its robust three-dimensional network structure (Figure 4e). The results of the time-sweep experiment indicate that the increase in |η*| clearly signifies an enhancement, solidification, or network formation within the material’s internal structure. Furthermore, both the drug-loaded and blank gels exhibit a storage modulus (G′) greater than their loss modulus (G″) (Figure 4e’).

### 2.2. Results of Transdermal Drug Release

Based on the comparative analysis of experimental data, CUR-M-DMNS-Gel exhibits a significantly enhanced delivery efficiency. The skin permeation rate at 48 h reaches (17.37 ± 0.36)%, which represents a 1.28-fold increase compared to CUR-M-Gel (13.60 ± 0.29)% and a 2.51-fold increase compared to CUR-M-DMNS (6.93 ± 0.55)%. Regarding skin retention performance, the retention rate of this composite system (62.41 ± 0.98)% is 1.29 times higher than that of CUR-M-Gel (48.51 ± 1.53)%, and 2.62 times higher than that of CUR-M-DMNS (23.85 ± 1.86)%. Kinetic studies indicate that this formulation achieves a total transdermal delivery rate of (79.78 ± 1.07)% through the spatiotemporal synergy between the microneedle channels and the gel matrix, characterized by an initial rapid penetration followed by a sustained release phase. This dual delivery mechanism not only improves the balance between transdermal delivery rate and duration of action but also generates notable synergistic effects through the functional complementarity among formulation components.

In this study, CUR at an excessive concentration was precisely weighed and delivered in the system. Following the addition of anhydrous ethanol, ultrasonic treatment and centrifugation were performed in succession. The supernatant was collected to prepare the test solution, with concentrations determined using a standard curve. The recorded CUR concentrations in the release medium were as follows: CUR-M-DMNs: 22.131 μg/g, CUR-M-Gel: 31.655 μg/g, and CUR-M-DMNs-Gel: 0.153 mg/g (*n* = 3). Additionally, the solubility of CUR in the release medium was noted as follows: CUR-M-DMNs: 125.239 μg/g, CUR-M-Gel: 167.815 μg/g, and CUR-M-DMNs-Gel: 0.682 mg/g (*n* = 3).

In summary, the ratios of CUR concentrations in the release medium to their corresponding maximum solubilities for the three formulations—CUR-M-DMNs, CUR-M-Gel, and CUR-M-DMNs-Gel—were found to be 17.67%, 18.86%, and 22.43%, respectively; all values strictly adhered to the required range of 10–30% under sink conditions. This finding indicates that drug concentration within the release medium remained consistently low throughout the experiment, thereby providing a stable core driving force for drug diffusion while effectively mitigating any interference from high drug concentrations on the release process itself; thus ensuring both accuracy and reliability of experimental data pertaining to transdermal drug release in this study. The materials used in the formulation and the rationale for their selection have been clearly outlined in the methods section of the abstract.

### 2.3. Analysis of the Results of Animal Model Construction

#### 2.3.1. Wound Healing Promotion of CUR-M-DMNs-Gel In Vivo

**Figure 5:**
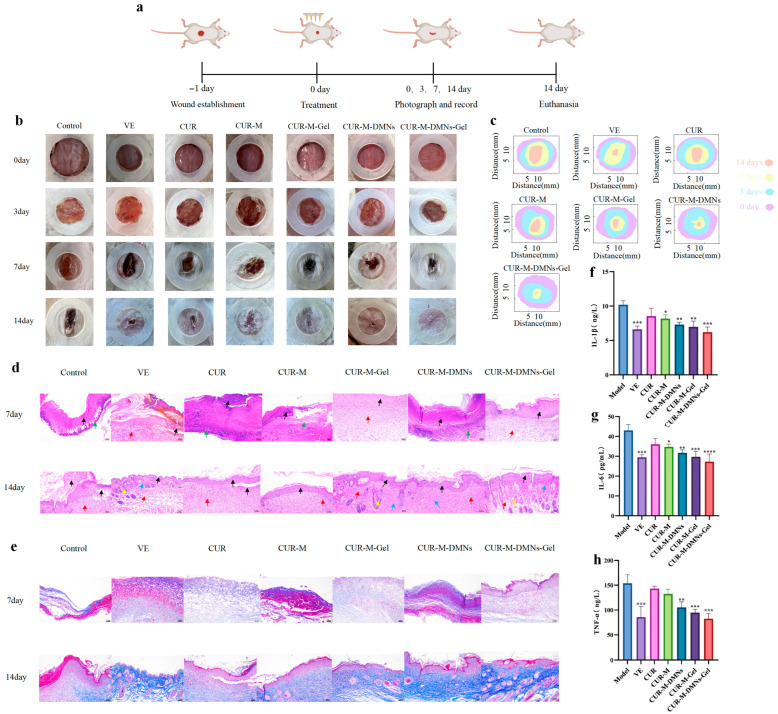
Therapeutic effects on mice with full-thickness skin defect models. (**a**) Schematic of the experimental timeline for skin wound healing. (**b**) Representative photographs of skin wounds (diameter: 1 cm) on days 0, 3, 7, and 14 post-treatment with different interventions. (**c**) Schematic diagram illustrating morphological changes in skin wounds across experimental groups. (**d**) Histological analysis by H&E staining. (**e**) Collagen deposition assessed by Masson’s trichrome staining. (**f**–**h**) Levels of inflammatory cytokines (IL-1β, IL-6, TNF-α). The asterisks in the figure indicate the statistical significance levels of the differences between groups: * *p* < 0.05, ** *p* < 0.01, *** *p* < 0.001, **** *p* < 0.0001.

To evaluate the in vivo therapeutic efficacy of CUR-M-DMNs-Gel, a full-thickness skin defect model was established to assess wound healing in mice. As illustrated in Figure 5a, the experimental groups received topical administration of vehicle (VE), CUR, CUR-M, CUR-M-DMNs, CUR-M-Gel, and CUR-M-DMNs-Gel, respectively. Wound images were captured on days 0, 3, 7, and 14 post-treatment. Compared with the Model group and other treatment groups, the CUR-M-DMNs-Gel group exhibited significantly accelerated wound-healing rates starting from day 3. This therapeutic advantage was sustained through day 7 and culminated in nearly complete wound closure with re-epithelialization observed by day 14 (Figure 5b). Changes in wound size over time were analyzed using simulation software (Figure 5c). The histological structures of wounds under different treatments were evaluated via H&E and Masson’s trichrome staining. H&E staining revealed intact epidermal structures and visible skin appendages in CUR-M-DMNs-Gel-treated wounds (Figure 5d). Masson’s trichrome staining demonstrated significantly enhanced collagen deposition in the CUR-M-DMNs-Gel treatment group compared to the control groups (Figure 5e). The study identified elevated expression levels of pro-inflammatory cytokines (IL-1β, IL-6, and TNF-α) in mice with full-thickness skin defects. Notably, the CUR-M-DMNs-Gel-treated group showed superior anti-inflammatory efficacy, as evidenced by marked reductions in these cytokine levels (Figure 5f–h).

#### 2.3.2. CUR-M-DMNs-Gel Ameliorates Ultraviolet Irradiation-Induced Skin Damage in Mice

**Figure 6:**
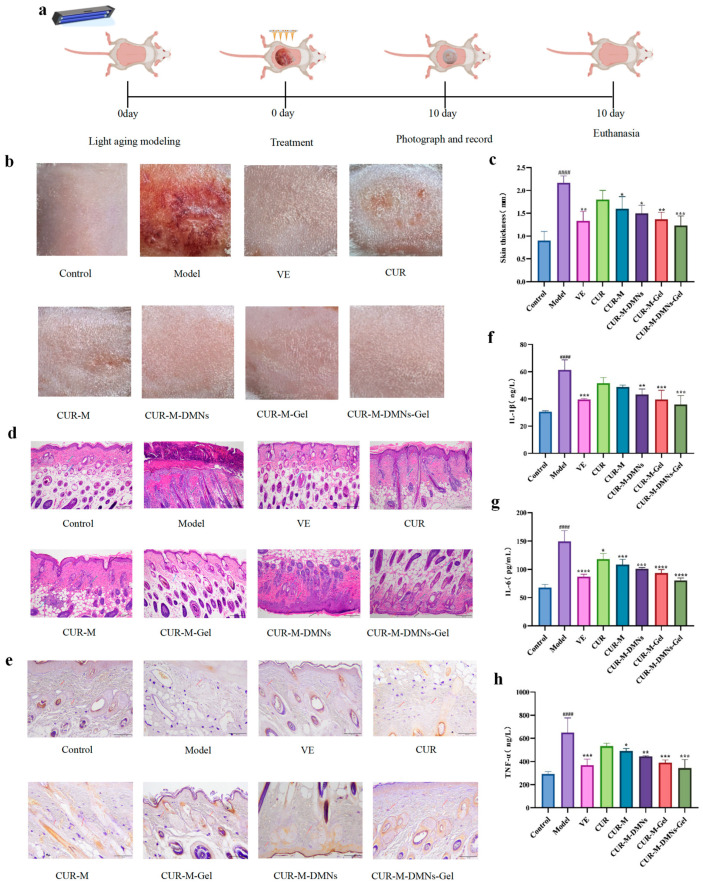
Therapeutic effects on photoaging mouse models. (**a**) Experimental timeline of photoaging induction and treatment. (**b**) Gross morphological observations of dorsal skin. (**c**) Measurement of skin thickness. (**d**) Histopathological analysis by H&E staining. (**e**) Elastic fiber visualization via aldehyde fuchsin staining. (**f**–**h**) Levels of inflammatory cytokines (IL-1β, IL-6, TNF-α) in skin homogenate supernatant. The asterisks in the figure indicate the statistical significance levels of the differences between groups: * *p* < 0.05, ** *p* < 0.01, *** *p* < 0.001, **** *p* < 0.0001. In the figure the statistical significance level of the differences between groups: #### indicates that compared with the model control group *p* < 0.0001.

This study evaluated the wound-healing efficacy in mice using a UV irradiation model. As shown in Figure 6a, experimental animals were divided into topical administration groups treated with VE, CUR, CUR-M, CUR-M-DMNs, CUR-M-Gel, and CUR-M-DMNs-Gel, respectively, to systematically compare the therapeutic effects of different formulations. Following UV exposure, the Model group exhibited significant erythema and epidermal scabbing on dorsal skin compared to the Control group. The CUR-M-DMNs-Gel group demonstrated nearly complete restoration of skin morphology to normal levels, characterized by a smooth and delicate epidermis without wrinkle formation (Figure 6b). The Model group exhibited a significant increase in dorsal skin thickness compared to the Control group. All treatment groups demonstrated varying degrees of reduction in skin thickness relative to the Model group, with the CUR-M-DMNs-Gel group showing the most pronounced therapeutic effect (Figure 6c). H&E staining revealed intact skin architecture and abundant skin appendages in the Control group, whereas the Model group displayed a marked reduction in skin appendages and extensive inflammatory cell infiltration. The CUR-M-DMNs-Gel group exhibited optimal restoration of skin structure, characterized by well-organized collagen fibers in the dermis (Figure 6d). Aldehyde fuchsin staining demonstrated densely distributed and orderly arranged elastic fiber structures in the dermis of the Control group. In contrast, the Model group exhibited disorganized and indistinct elastic fiber architecture, whereas the CUR-M-DMNs-Gel group maintained integrated elastic fiber structures (Figure 6e). The photoaged mouse model demonstrated significantly elevated expression levels of pro-inflammatory cytokines IL-1β, IL-6, and TNF-α. Notably, the CUR-M-DMNs-Gel treatment group displayed the most potent anti-inflammatory effects among all interventions (Figure 6f–h).

### 2.4. Discussion

This study successfully developed CUR-M-DMNs-Gel by integrating micelle technology, dissolving microneedles, and sustained-release gel formulations. This multi-faceted technological approach effectively addresses the inherent pharmaceutical limitations of CUR, including its poor water solubility, instability, and limited transdermal bioavailability. Consequently, it provides an innovative technical platform for the transdermal delivery of natural products.

Current research indicates that while single delivery systems have enhanced drug delivery efficiency to some extent, their clinical application remains constrained by short duration of action and limited penetration depth. The synergistic combination of three technologies developed in this study not only significantly improves the solubility and stability of CUR but also facilitates rapid transdermal penetration and sustained release of the drug within skin tissue.

The design of the CUR-M-DMNs-Gel system exemplifies an innovative integration of multi-level delivery strategies. The core layer consists of CUR-loaded polymer micelles, which are self-assembled from amphiphilic block copolymers and exhibit a typical core–shell structure. The hydrophobic core effectively encapsulates CUR molecules, while the hydrophilic shell ensures stable dispersion of the micelles in aqueous environments. This nanostructure not only significantly enhances the apparent solubility of CUR but also protects the drug from hydrolysis and photodegradation through steric hindrance effects. The middle layer features a soluble microneedle array, fabricated using a layer-by-layer casting method. This design facilitates effective penetration through the stratum corneum without engaging pain receptors located in the dermis. The concentration of CUR-loaded micelles within the microneedles has been optimized to ensure rapid dissolution in interstitial fluid within skin tissue; subsequently, released micelles diffuse into deeper tissues via appendage pathways such as hair follicles and sweat glands. The outer layer comprises a thermosensitive gel matrix. This property of in situ gelation not only simplifies application but also establishes a persistent adhesive drug reservoir on the skin surface, enabling continuous drug release for painless administration and rapid therapeutic effect. Concurrently, this gel matrix—with its adhesive characteristics and sustained-release capabilities—prolongs treatment duration, thereby establishing a dual drug delivery mechanism. This innovative design demonstrates exceptional skin retention and transdermal efficiency.

In animal model studies, the CUR-M-DMNs-Gel system exhibited significant therapeutic advantages. In full-thickness skin defect models, the treatment group demonstrated a markedly higher wound-healing rate on day 14 compared to the single-technology groups. Histological analysis revealed that this composite system could substantially enhance epidermal regeneration, increase collagen deposition, and improve angiogenesis. Investigations into molecular mechanisms indicated that this system effectively down-regulated the expression of inflammatory factors such as TNF-α, IL-6, and IL-1β. In skin photoaging models, CUR-M-DMNs-Gel treatment significantly alleviated symptoms of photodamage, thereby reducing oxidative stress and collagen degradation.

However, despite the promising therapeutic effects observed in vitro and in animal studies, clinical translation still encounters challenges including complex preparation processes and long-term safety verification. Future research should prioritize optimizing compatibility between microneedles and gels to streamline manufacturing processes while conducting mechanistic studies through cellular experiments. Furthermore, exploring applications for complex skin injuries such as chronic ulcers and scar repair can broaden the therapeutic potential of this system, ultimately providing more comprehensive treatment strategies within dermatology.

## 3. Conclusions

In summary, a novel nanotechnology-based composite drug delivery system (CUR-M-DMNs-Gel) was successfully developed to enhance wound healing and mitigate skin damage, particularly in conditions associated with photoaging. The system was initially designed by formulating CUR into CUR-M, which significantly improved drug solubility, stability, and transdermal permeability. To overcome the limitations imposed by the stratum corneum barrier, CUR-M-DMNs were subsequently fabricated to enable painless penetration and rapid drug release through microneedle dissolution, thereby establishing efficient cutaneous delivery pathways. Concurrently, a sustained-release CUR-M-Gel was developed to prolong the therapeutic effect. This gel formulation not only facilitated prolonged drug retention on the skin surface but also ensured stable anchoring of CUR-M-DMNs due to its excellent adhesive properties. Ultimately, a synergistic nanocomposite drug delivery system was constructed by embedding CUR-M-DMNs within the CUR-M-Gel matrix. The CUR-M-DMNs enabled targeted drug delivery via physical barrier penetration, while the CUR-M-Gel provided extended therapeutic activity through a dual sustained-release mechanism. Preclinical animal studies demonstrated significant therapeutic efficacy in both full-thickness skin defect and photoaging murine models. This study presents an innovative therapeutic strategy that integrates high delivery efficiency with prolonged action for the treatment of skin injuries.

## 4. Materials and Methods

### 4.1. Materials

#### 4.1.1. Reagents

CUR (Macklin Biochemical Co., Ltd., Shanghai, China), absolute ethanol (Yonghua Chemical Co., Ltd., Changshu, Suzhou, Jiangsu Province, China), vitamin E polyethylene glycol succinate (TPGS, Xi’an Ruixi Biological Technology Co., Ltd., Xi’an, China), PVP K30, PVP K90 (Shanghai Maokang Biotechnology Co., Ltd., Shanghai, China), NP-700, aluminum chloride, glycerol, tartaric acid (Shanghai Yuanye Biotechnology Co., Ltd., Shanghai, China), hyaluronic acid (HA, Bloomage Biotechnology Co., Ltd., Jinan, China), and isoflurane (Hebei Jindafu Pharmaceutical Co., Ltd., Xingtai, China).

The equipment used in this experiment includes: 78-1 type magnetic heating stirrer produced by Jiangnan Experimental Instrument Factory, Changzhou, China; NewClassic MS series electronic balance of Mettler Toledo (produced in Shanghai, China), model MS104TS; JK-300B type ultrasonic cleaner produced by Jinike Machinery Manufacturing Co., Ltd., Hefei, China; Synergy2 multi-mode microplate reader of Molecular Devices (assembled in Shanghai, China), model Synergy H2; RE-201D type rotary evaporator produced by Shanghai Yarong Biochemical Instrument Factory; TGL-16B type high-speed centrifuge produced by Shanghai Ziqiao Experimental Equipment Co., Ltd., Shanghai, China; DHG-9070A type electric heating blower drying oven produced by Shanghai Bosun Medical Biological Instrument Co., Ltd., Shanghai, China; MX-PDMS-100 type soluble microneedle polydimethylsiloxane (PDMS) master mold custom produced by Taizhou Weixin Medical Technology Co., Ltd., Taizhou, China; PAT-2000 type initial adhesion tester of Pantone Color Card Company, Carlstadt, NJ, USA; HY-ADH-500 type retention adhesion tester produced by Shanghai Haiyou Experimental Equipment Co., Ltd., Shanghai, China; SU8010 type scanning electron microscope (SEM) of Hitachi, Tokyo, Japan; Tecnai G2 F20 type transmission electron microscope (TEM) of FEI Company, Hillsboro, OR, USA; Axio Imager A2 type optical microscope of Zeiss, Oberkochen, Germany; AR2000 type rheometer of TA Instruments, New Castle, DE, USA; Infinite 200 Pro type enzyme-linked immunosorbent assay (ELISA) reader of Tecan, Männedorf, Switzerland.

#### 4.1.2. Animals

This study was approved by the Animal Ethics Committee of Changchun University of Traditional Chinese Medicine (Approval No.: 2025052), with the approval date set for 4 March 2025. Throughout the entire experimental process, every effort was made to minimize the animals’ suffering. For this research, female Kunming strain mice weighing between 28 and 30 g (KM grade, Certificate No.: SCXK (Liao) 2020-0001) were selected to evaluate the wound-healing effects of CUR-M-DMNs-Gel.

This study complies with national laws, regulations, technical standards, and relevant provisions governing the management of experimental animals and animal experiments. Qualified animals possessing appropriate animal use licenses were housed in barrier environment facilities and had unrestricted access to food and water 24 h a day. Animal feed, bedding, and drinking water underwent disinfection and sterilization prior to use. Cages and bedding were regularly cleaned and replaced. A skin excision was performed on the backs of mice to establish a skin injury model. The experimental groups received topical administration of vehicle (VE), CUR, CUR-M, CUR-M-DMNs, CUR-M-Gel, and CUR-M-DMNs-Gel. Six mice per group were utilized for the study, with drug administration occurring daily. During the experiment, mice were anesthetized using isoflurane before applying the corresponding treatment drugs to the affected area. The treatments were changed once daily over a two-week period. Skin injury sites were monitored on a daily basis. Blood samples, serum specimens, organ tissues, and skin tissues were collected for pathological section analysis. Method of euthanasia: Following completion of the experiment, animals were euthanized via carbon dioxide asphyxiation. The carcasses were subsequently frozen and treated as medical waste for unified disposal by the institution.

### 4.2. Methods

#### 4.2.1. Preparation of CUR-M, CUR-M-DMNs and CUR-M-Gel

##### CUR-M

CUR micelles were prepared using the film dispersion method. A suitable amount of CUR and TPGS was weighed into a beaker, followed by the addition of ethanol. The mixture was sonicated for 10 min to ensure complete dissolution, then transferred to a flask where excess ethanol was removed via rotary evaporation under reduced pressure for 1 h. Subsequently, the flask was placed in a vacuum desiccator at room temperature overnight. Afterward, 8 mL of distilled water was added and stirred at a specific temperature for 10 min to hydrate the solution. The mixture was then centrifuged at 4 °C and 10,000 rpm for 10 min; the supernatant obtained constituted the CUR-M. To prepare freeze-dried powder, CUR-M was mixed with 10% mannitol and frozen at −80 °C before being subjected to freeze-drying.

##### CUR-M-DMNs

A homogeneous solution containing 20% PVP K30 and 20% HA as needle tip solution was prepared from CUR-M and allowed to stand at room temperature until no bubbles formed. Additionally, another uniform mixture consisting of 20% PVP K30 and 20% PVP K90 served as backing solution. The needle tip solution was poured into square PDMS molds and centrifuged at 4000 rpm for 10 min; this process was repeated after flipping the mold by 180°. Following centrifugation, any residual needle body solution on the surface of the microneedle mold was scraped off. After drying in a desiccator for an additional 15 min, backing solution equal to the edge height of microneedle molds was added, before subjecting it again to centrifugation at 3500 rpm for 5 min and flipping it 180° under identical conditions. The resulting molds were dried in a desiccator until they could be demolded easily; thus yielding CUR-M-DMNs.

##### CUR-M-GEL

The thickening agent PVP K30 dissolved in micelles along with crosslinking agent tartaric acid were uniformly mixed together as phase A. Glycerin was measured out separately while sequentially adding aluminum oxide and NP-700 to form phase B through thorough mixing. After ensuring both phases A and B were well combined through stirring over time, the resultant phase A would be poured into phase B allowing them to mix homogeneously. Upon completion of gel formation, a glass rod would coat this gel onto the backing layer which is subsequently covered with an anti-adhesive layer, resulting in gel preparation.

#### 4.2.2. Characterization of CUR-M, CUR-M-DMNs and CUR-M-Gel

##### Characterization of CUR-M

The Tyndall effect of CUR-M was observed by directing a red laser beam from one side. The morphology of CUR-M was examined using transmission electron microscopy (TEM). Particle size and zeta potential were analyzed with a Malvern laser particle size analyzer. TEM: The instrument model is the Tecnai G2 F20 from FEI Company, Hillsboro, OR, USA. The morphology of CUR-M was observed under a transmission electron microscope (TEM) at an accelerating voltage of 100 kV. To prepare the TEM samples, a drop of micelle solution was placed on a copper grid and stained with phosphotungstic acid solution (2%) for approximately 15 s. Subsequently, the samples were allowed to dry slowly in air before being examined under the TEM.

Encapsulation efficiency (EE) and drug loading content (DL) were determined through high-speed centrifugation. Furthermore, these parameters were also assessed following dissolution of the carriers in ethanol, followed by ultraviolet–visible spectrophotometric analysis. EE and DL were calculated according to Equations (1) and (2), respectively.

(1) $$EE(\%)=\frac{m_{abs}}{m_{t}}\times 100\%$$

(2) $$DL(\%)=\left[\frac{m_{abs}}{\left(m_{abs}+m_{v}\right)}\right]\times 100\%$$

where m_abs_ stands for the drug dosage in micelles, m_v_ for carrier dosage, and m_t_ for drug delivery dosage.

Fourier-transform infrared (FTIR) spectroscopy was employed to characterize CUR, blank micelle lyophilized powder, and CUR-M across a wavenumber range of 4000–400 cm^−1^.

X-ray diffraction (XRD) analysis was performed using a Bruker D8 Advance diffractometer within a scanning angle range of 5.0–45.0° at a scan rate of 2° min^−1^. All measurements were conducted at room temperature, with triplicate testing performed for each sample.

##### Characterization of CUR-M-DMNs

Following the drying and demolding processes, the macroscopic morphology of the samples was initially assessed using an optical microscope at 10× magnification. For ultrastructural characterization, CUR-M-DMNs specimens were subjected to gold sputter coating prior to surface microstructural analysis by scanning electron microscopy (SEM).

To evaluate the mechanical strength of CUR-M-DMNs, the microneedles were placed at the center of a flat stainless steel plate and subjected to analysis using a universal testing machine. A flat-headed stainless steel probe (diameter: 0.5 cm) was programmed to advance toward the CUR-M-DMNs at a constant speed of 0.5 mm/min, with the force-displacement curve recorded simultaneously.

A penetration test was carried out using six-layer Parafilm membranes to simulate skin tissue. Under a constant force of 20 N applied for 60 s, the membranes were manually separated layer by layer to assess micropore formation, with photographic documentation captured at each stage. A hole formation ratio versus insertion depth curve was subsequently generated for CUR-M-DMNs.

The CUR-M-DMNs were inserted into the skin and then removed at 0, 4, 7, and 10 min for optical microscopic imaging. In parallel, CUR-M-DMNs were applied to ex vivo mouse skin tissue. The tissue was embedded in paraffin and stained with hematoxylin and eosin (H&E) to visualize the micropores formed by the microneedles.

The CUR-M-DMNs were vertically applied to the treated skin surface and removed at 0, 5, 10, and 15 min. Digital photographs were captured to document morphological alterations of the skin, with particular attention to the closure dynamics of the puncture sites.

The CUR-M-DMNs patches were inserted into the dorsal skin of mice and retained for 5 min prior to removal. The treatment sites were digitally photographed at 0 h, 0.5 h, 1 h, 6 h, and 24 h post-removal to evaluate potential dermal edema formation.

##### Characterization of CUR-M-Gel

The morphological characteristics of the blank gel and CUR-M-Gel were visually examined at room temperature.

To evaluate the adhesion properties of CUR-M-Gel, the formulation was applied onto the surfaces of various substrates (including skin, articular models, plastics, glass, cast iron, and stainless steel), followed by analysis of its adhesive strength and stability.

The CUR-M-Gel samples were divided into three equal portions (*n* = 3) and placed in open beakers for the experiment under ambient temperature conditions (25 ± 1 °C). Following each experimental cycle, the mass variation in the CUR-M-Gel was quantitatively determined using a precision electronic analytical balance.

In a separate set of experiments, the CUR-M-Gel samples were evenly divided into three portions (*n* = 3) and individually immersed in beakers containing deionized water at room temperature. After each immersion cycle, the samples were carefully retrieved, gently blotted with filter paper to remove residual surface moisture, and further dried with clean filter paper prior to immediate weight measurement.

The rheological properties of CUR-M-Gel were evaluated at 37 °C using a rheometer fitted with an 18 mm stainless steel parallel plate. Frequency sweep tests ranging from 0.1 to 100 rad/s were conducted under a constant strain of 1% to assess the frequency dependence of the storage modulus (G′) and loss modulus (G″). The structural stability of the gel was determined by analyzing the variation patterns of the dynamic moduli as well as the relationship between G′ and G″. A time-scan experiment was also conducted to monitor the changes in G′ and G″ over a period of 0–60 min at 37 °C, in order to assess the stability.

#### 4.2.3. Transdermal Drug Release

The formulations (CUR-M-Gel, CUR-M-DMNs, and CUR-M-DMNs-Gel) were topically applied to excised skin samples mounted in Franz diffusion cells. The receptor compartment was filled with phosphate-buffered saline (PBS) containing 1% (*w*/*v*) Tween 80 and 20% (*v*/*v*) ethanol, and the system was maintained at a constant temperature of 37 ± 0.5 °C. At predetermined time intervals, 1 mL aliquots were withdrawn from the receptor medium. Upon completion of the experiment, the skin tissues were homogenized to assess the cumulative transdermal drug permeation rate (%).

Franz Diffusion Cell: The RYJ-12B transdermal drug diffusion testing instrument, produced by Shanghai Huanghai Pharmaceutical Testing Instrument Co., Ltd. Shanghai, China, is designated as model RYJ-12B. The country of origin is China, and the volume of the receptor chamber is 8 mL. This study was conducted under equilibrium conditions.

#### 4.2.4. Animal Model Construction

##### Establishment of a Clinical Wound-Healing Model

Following a one-week acclimatization period, the mice were randomly assigned to seven experimental groups (*n* = 6 per group): Control, VE, CUR, CUR-M, CUR-M-Gel, CUR-M-DMNs, and CUR-M-DMNs-Gel. Under isoflurane anesthesia, a circular full-thickness skin wound (1 cm in diameter) was surgically created on the dorsal region of each mouse. Digital images of the wounds were captured and subjected to morphometric analysis on postoperative days 0, 3, 7, and 14.

Mice were euthanized on postoperative days 7 and 14. Full-thickness skin specimens from the dorsal wound areas were carefully excised and fixed in a 4% paraformaldehyde solution for 24 h. Following fixation, the samples underwent dehydration through a graded ethanol series, clearing in xylene, and embedding in paraffin blocks. Serial sections (5 μm in thickness) were subsequently prepared using a rotary microtome for histological evaluation, including H&E staining and Masson’s trichrome staining.

Blood samples were allowed to clot at 4 °C for a period of 2 h and were subsequently centrifuged at 3000 revolutions per minute (rpm) for 15 min to separate the serum. The concentrations of interleukin-1β (IL-1β), interleukin-6 (IL-6), and tumor necrosis factor-alpha (TNF-α) in the serum were measured using commercially available enzyme-linked immunosorbent assay (ELISA) kits, in accordance with the manufacturer’s instructions.

##### Establishment of Photoaging Mouse Model

Following a one-week acclimatization period, the mice were randomly assigned to eight experimental groups (*n* = 6 per group): Control, Model, VE, CUR, CUR-M, CUR-M-Gel, CUR-M-DMNs, and CUR-M-DMNs-Gel. The photoaging model was induced by ultraviolet (UV) irradiation at a fixed distance of 10 cm. The irradiation protocol consisted of an initial adaptive exposure of 2 h on Day 1, followed by daily exposure of 4 h from Day 2 to Day 4, and subsequently extended to 6 h per day from Day 5 to Day 10. Morphological changes in the dorsal skin were recorded through digital photography and subjected to morphometric analysis on a daily basis throughout the experimental period.

Dorsal skin tissue samples were surgically excised from mice in each experimental group. Following collection, full-thickness skin specimens were carefully flattened to maintain their native morphology while preventing mechanical deformation. The thickness of the dorsal skin was accurately measured for each specimen using a vernier caliper.

Skin samples were collected from the shaved dorsal skin of mice euthanized at the conclusion of day 10. Following a 24 h fixation period in paraformaldehyde, the specimens were embedded in paraffin and sectioned. Histological analysis was conducted using hematoxylin and eosin (H&E) staining and aldehyde fuchsin staining.

The dorsal skin of mice was excised, and connective tissues were carefully removed. Following rinsing with saline and blotting dry with filter paper, the tissue was precisely weighed and homogenized in saline at a 1:9 (*w*/*w*) ratio. The resulting homogenate was centrifuged at 4500 revolutions per minute (r/min) for 15 min to obtain the supernatant. The concentrations of interleukin-1β (IL-1β), interleukin-6 (IL-6), and tumor necrosis factor-alpha (TNF-α) were quantified in accordance with the manufacturer’s instructions provided with the assay kits.

##### Sample Size and Administration of Medication

According to the 3R principle, the sample size of *n* = 6 per group was determined a priori using a power analysis conducted with G*Power software (version 3.1.9.7). This study expected a large effect size (Cohen’s d = 1.8). With a significance level (α) set at 0.05 and a desired power (1-β) of 0.8 for a two-tailed independent *t*-test, the analysis indicated that 6 animals per group would be sufficient to detect a statistically significant effect.

The VE group received 1 mg/mL (0.1 mL) daily, the CUR group received 1 mg/mL (0.1 mL), the CUR-M group was administered 0.4 mL, the CUR-M-Gel group received 0.1 mg per patch, and the CUR-M-DMNs group was given one piece of treatment. The CUR-M-DMNS-Gel group received a combination of CUR-M-Gel and CUR-M-DMNS. Treatment for the wound model lasted for 14 days, while treatment for the photodamage model continued for 10 days, with dressing changes performed once daily.

### 4.3. Statistical Analysis

All results presented in this study are expressed as mean ± standard deviation (SD). Statistical analysis was conducted using a two-tailed Student’s *t*-test or one-way analysis of variance (ANOVA) in Excel. *p* < 0.05 was considered statistically significant. The levels of significance were indicated as * *p* < 0.05, ** *p* < 0.01, and *** *p* < 0.001.

## Figures and Tables

**Table 1:** Stability study results of CUR-M (*n* = 3).

Time (day)	Appearance	EE (%)	DL (%)
0	Clear and transparent	95.72 ± 0.93	5.58 ± 1.29
3	Clear and transparent	95.54 ± 0.08	5.49 ± 1.13
7	Clear and transparent	95.18 ± 1.26	5.43 ± 0.95
14	Clear and transparent	94.83 ± 0.76	5.39 ± 0.59
21	Clear and transparent	94.64 ± 1.06	5.33 ± 1.72

## Data Availability

The original contributions presented in this study are included in the article/Supplementary Materials. Further inquiries can be directed to the corresponding authors.

## Supplementary Materials

The following supporting information can be downloaded at: https://www.mdpi.com/article/10.3390/gels11090727/s1, Figure S1: UV Spectra of CUR and TPGS; Figure S2: UV Standard Curve of CUR; Table S1: Results of Daytime Precision Experiment (±s, *n* = 3); Table S2: Results of Intraday Precision Experiments (±s, *n* = 3); Table S3: Results of Reproducibility Tests (±s, *n* = 5); Table S4: Spiking Recovery Rate Experiment Results (±s, *n* = 3); Table S5: Results of CUR-M Investigation (±s, *n* = 3); Table S6: Effect of Different TPGS to CUR Mass Ratios on EE and DL of CUR-M (*n* = 3); Table S7: Scoring Table for Microneedle Matrix Selection (±s, *n* = 3); Table S8: Effect of different matrix formulations on the puncture capability of CUR-M-DMNs (*n* = 3); Table S9: The Impact of Different Mass Fractions on the Comprehensive Evaluation of Gel Quality (*n* = 3); Table S10: Effects of Different Matrix Mass Fractions on Initial and Sustained Adhesive Strengths of CUR-M-Gel (*n* = 3).
