# Supplementary material for: Preparation of Curcumin Nanocomposite Drug Delivery System and Its Therapeutic Efficacy on Skin Injury
Source: Gels. 2025 Sep 11;11(9):727. doi: 10.3390/gels11090727 (PMC12469627; doi:10.3390/gels11090727)
Supplement: Supplementary file 1 [file gels-11-00727-s001.zip › gels-3805058-supplementary.pdf]

Supplementary Materials

S1. A validated analytical method for the quantitative determination of curcumin

S1.1. Determination of the Maximum Absorption Wavelength of CUR

The UV spectra of TPGS and CUR within the wavelength range of 270 nm to 600 nm are illustrated in Figure S1. The maximum absorption wavelength of CUR is identified at 426 nm, while TPGS exhibits no ultraviolet absorption at this wavelength. This indicates that TPGS does not interfere with the measurement of CUR at 426 nm, demonstrating a high specificity for CUR under this analytical method. Consequently, in subsequent experiments, the detection wavelength for the analysis of CUR content was selected as 426 nm.

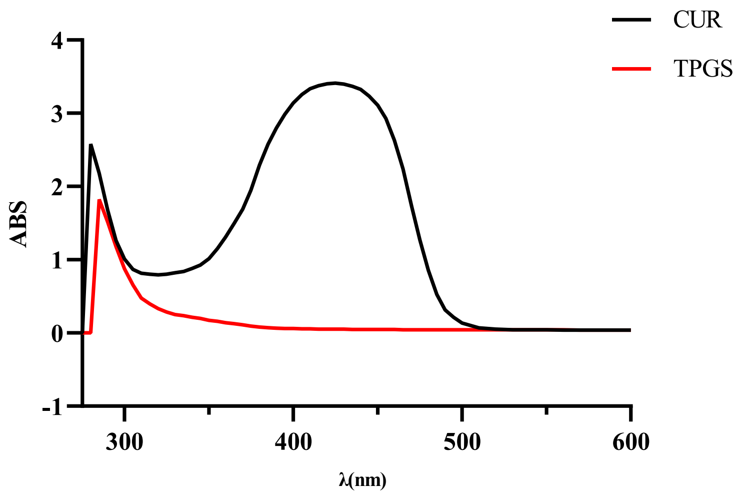

Figure S1. UV Spectra of CUR and TPGS.

S1.2. Establishment of the CUR Standard Curve

The standard curve for CUR was constructed by plotting the concentration of CUR (C) on the x-axis and the absorbance values (A) on the y-axis, as illustrated in Figure S2. The corresponding linear equation is as follows:

$$Y=0.0814X+0.0677，r=0.9993 \qquad (S1)$$

The results indicate that CUR exhibits a strong linear relationship with absorbance within the concentration range of 0.5 to 32 μg/mL, yielding an r value of

0.9993, which meets the methodological requirements.

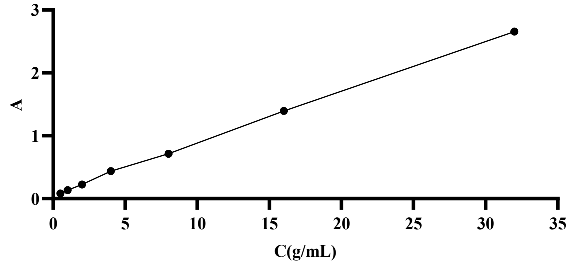

Figure S2. UV Standard Curve of CUR.

S1.3. Daytime Precision Testing

The results of the intra-day precision are presented in Table S1. Analysis of the data from this table indicates that the RSD values corresponding to three different concentrations are all less than 3%. This demonstrates that the method for content analysis exhibits good intra-day precision, thereby meeting methodological requirements.

Table S1: Results of Daytime Precision Experiment (±s, n=3)

concentration（μg/mL）	average value±SD	RSD
0.5	0.290±0.007	2.44%
4	3.685±0.058	1.58%
32	29.942±0.471	1.57%

S1.4. Intraday Precision Testing

The results of the intraday precision are presented in Table S2. From the data analysis, it can be observed that the RSD values corresponding to three different concentrations are all less than 3%. This indicates that the method for content analysis demonstrates good intraday precision and meets methodological requirements.

Table S2: Results of Intraday Precision Experiments (±s, n=3).

concentration（μg/mL）	average value±SD	RSD
0.5	0.320±0.006	1.92%
4	3.781±0.015	0.42%
32	30.353±0.155	0.51%

S1.5. Reproducibility Testing

The results of the reproducibility tests are presented in Table S3. Analysis of the data from the table indicates that the relative standard deviation (RSD) for absorbance values at three different concentrations is less than 3%. This demonstrates that the precision of the content analysis method within a single day is satisfactory and meets methodological requirements.

Table S3: Results of Reproducibility Tests (±s, n=5).

concentration（μg/mL）	average value±SD	RSD
0.5	0.511±0.007	1.39%
4	5.376±0.039	0.73%
32	35.946±0.095	0.27%

S1.6. Sample Recovery Rate Experiment

The results of the spiking recovery rate experiment are presented in Table S4. Analysis of the data indicates that the RSD for all three concentrations is less than 3%. This demonstrates that the analytical method employed for content determination exhibits a good spiking recovery rate, confirming its accuracy and reliability.

Table S4. Spiking Recovery Rate Experiment Results (±s, n=3).

concentration（μg/mL）	average value±SD	RSD
0.5	99.84%±0.007	1.42%
4	102.36%±0.056	1.37%
32	100.01%±0.104	0.33%

S2. Material screening and performance optimization of CUR-M, CUR-M-DMNs, and CUR-M-Gel

S2.1. Optimization of the CUR-M formulation and screening of encapsulation performance.

According to the results of EE and DL, as shown in the table, the optimal preparation scheme for CUR-M has been determined to be a drug-to-excipient ratio of 17:1, with a hydration temperature of 35°C, a hydration time of 10 minutes, and a hydration volume of 8 ml(Table S5).

Table S5. Results of CUR-M Investigation (±s, n=3).

Drug excipient ratio	EE（%）	DL（%）
10:1	44.60±2.34	4.39±0.33
13:1	58.08±1.2	4.55±0.26
15:1	73.62±2.32	4.71±0.1
17:1	94.73±0.5	5.6±0.02
20:1	93.16±0.66	4.64±0.48
**Hydration temperature（℃）**	**EE（%）**	**DL（%）**
30	86.62±1.54	4.89±0.2
35	95.18±0.37	5.31±0.15
40	76.85±2.83	4.21±0.31
45	67.94±2.83	3.77±0.51
50	65.26±2.92	3.72±0.26
**Hydration time(min)**	**EE（%）**	**DL（%）**
5	92.05±2.11	5.56±0.07
10	94.99±0.15	5.64±0.02
15	91.44±1.27	5.43±0.07
20	80.02±1.28	4.08±0.06
30	61.88±0.79	3.54±0.05
**Hydration volume(ml)**	**EE（%）**	**DL（%）**
4	59.21±1.65	3.31±0.13
6	80.00±2.60	4.55±0.03
8	95.57±0.34	5.43±0.03
10	69.96±2.13	4.01±0.24
12	60.22±2.63	3.67±0.30

To investigate the optimal TPGS-based micellar formulation for CUR encapsulation, ten batches of CUR-M were prepared by varying the mass ratio of TPGS to CUR. The EE and DL were subsequently assessed.

Optimization of the CUR-M formulation and evaluation of encapsulation

performance revealed that Prescription 5 demonstrated superior drug loading and encapsulation characteristics, achieving an EE of 94.99 ± 0.15% and a DL of 5.64 ± 0.02%. Consequently, this formulation was selected as the final optimized version (Table S6).

Table S6. Effect of Different TPGS to CUR Mass Ratios on EE and DL of CUR-M (n=3).

Formulation	EE（%）	DL（%）
1	73.62±2.32	4.71±0.10
2	93.16±0.66	4.64±0.48
3	86.62±1.54	4.89±0.20
4	76.85±2.83	4.21±0.31
5	94.99±0.15	5.64±0.02
6	91.44±1.27	5.43±0.07
7	80.02±1.28	4.08±0.06
8	80.00±2.60	4.55±0.03
9	69.96±2.13	4.01±0.24
10	60.22±2.63	3.67±0.30

S2.2. Screening of CUR-M-DMNs Substrates and Evaluation of Puncture Performance.

The data presented indicate that it is challenging to achieve ideal DMNs using a single type of material. When HA is used alone as the matrix, the resulting DMNs exhibit insufficient mechanical strength while possessing excessive toughness. Conversely, when PVP K30 is utilized solely as the matrix, the mechanical strength becomes excessively high, leading to brittleness and inadequate toughness. Therefore, we propose employing a solution consisting of 20% PVP K30 and 20% HA, which will be thoroughly mixed to serve as the needle tip solution. Additionally, a mixture of 20% PVP K30 and 20% PVP K90 will be prepared uniformly to function as the backing solution. It can be observed that after compounding these materials, the overall performance rating of the resulting DMNs is significantly improved, effectively addressing issues related to demolding difficulty and low needle density(Table S7).

Table S7. Scoring Table for Microneedle Matrix Selection (±s, n=3)

Serial Number	Needle Tip Matrix	Backing Matrix	Formability	Puncturability	Flexibility	Total Score
1	20%HA	20%PVPK30＋20%PVPK90	10	7.33	10	27.33
2	20%PVPK30	20%PVPK30＋20%PVPK90	9	9	10	28

3	20%PVPK90	20%PVPK30＋20%PVPK90	10	7.67	10	27.67
4	20%HA＋20%PVPK30	20%PVPK30＋20%PVPK90	10	10	9.67	29.67
5	20%HA＋20%PVPK90	20%PVPK30＋20%PVPK90	9.67	8.67	10	28.33
6	20%HA＋20%PVPK30	15%PVA	10	10	4	24.67
7	20%HA＋20%PVPK30	25%PVA	10	10	6	26
8	20%HA＋20%PVPK30	20%PVPK30	10	10	6.33	26.33
9	20%HA＋20%PVPK30	30%PVPK30	10	10	6	25
10	20%HA＋20%PVPK30	10%PVPK90	10	10	7	26
11	20%HA＋20%PVPK30	20%PVPK90	10	10	7.67	27
12	20%HA＋20%PVPK30	30%PVPK90	10.00	10.00	7.67	27.67

Following the preparation based on various matrix formulations, the performance of five batches of CUR-M-DMNs samples was assessed through puncture tests. The results indicated that Prescription 4 achieved a puncture capability of 100%, demonstrating its exceptional mechanical penetration performance. Consequently, it was selected as the final optimized prescription (Table S8).

Table S8. Effect of different matrix formulations on the puncture capability of CUR-M-DMNs (n=3).

Formulation	Penetration Capability
1	73.33%
2	76.67%
3	86.67%
4	100%
5	90.00%

S2.3. Optimization and Screening of Adhesive Properties of CUR-M-Gel

According to the results of the comprehensive evaluation of gel quality based on the varying mass fractions of its components, as illustrated in the table, we have determined that the optimal formulation consists of 6% NP-700, 0.1% aluminum chloride, 3% PVP K30, and 0.3% tartaric acid (Table S9).

Table S9. The Impact of Different Mass Fractions on the Comprehensive Evaluation of Gel Quality (n=3)

Quality Fraction of NP-700	Initial adhesion	Residual adhesion	Sensory evaluation	Total score
2%	23.35	13.65	37.00	74
4%	23.67	8.22	37.67	69.56
6%	30.00	30.00	39.67	99.67

8%	24.67	28.36	38.33	91.36
10%	23.67	16.75	38.33	78.75
**Quality Fraction of aluminum chloride**	**Initial adhesion**	**Residual adhesion**	**Sensory evaluation**	**Total score**
0.04%	21.17	25.69	35.67	82.53
0.10%	30.00	30.00	39.67	99.67
0.16%	17.81	10.03	37.33	65.17
0.20%	21.17	9.36	36.33	66.86
0.24%	21.28	8.15	35.00	64.43
**Quality Fraction of PVPK30**	**Initial adhesion**	**Residual adhesion**	**Sensory evaluation**	**Total score**
2%	22.44	21.31	37.33	81.08
3%	30.00	30.00	39.33	99.33
4%	21.44	25.98	38.33	85.75
5%	21.44	8.04	37.67	67.15
6%	18.22	6.93	36.67	61.82
**Quality Fraction of tartaric acid**	**Initial adhesion**	**Residual adhesion**	**Sensory evaluation**	**Total score**
0.10%	25.42	20.99	37.67	84.08
0.20%	25.42	26.44	38.67	89.53
0.30%	30.00	30.00	39.67	99.67
0.40%	21.94	13.16	37.67	72.77
0.50%	18.47	24.67	36.67	79.81

In this study, ten batches of CUR-M-Gel were prepared by varying the matrix mass fraction. The initial adhesion (measured by ball number) and sustained adhesion (measured in seconds) of each batch were comprehensively evaluated.

Formulation 3 demonstrated optimal initial adhesion (No. 9 steel ball) and sustained adhesion (lasting 18 seconds), thereby confirming its superior adhesive properties. Consequently, it was selected as the ultimately optimized formulation (Table S10).

Table S10. Effects of Different Matrix Mass Fractions on Initial and Sustained Adhesive Strengths of CUR-M-Gel (n = 3)

Formulation	Initial Tack (Ball No.)	Adhesive Holding Time (s)
1	7	8
2	7	5
3	9	18
4	5	5
5	6	5
6	7	17
7	7	15
8	7	5
9	6	7
10	5	14
